# Supplementary material for: Increased Rate of Yeast Cultivation from Packaged Beer with Environmentally Relevant Anaerobic Handling
Source: Microbiol Spectr. 2022 Oct 31;10(6):e02656-22. doi: 10.1128/spectrum.02656-22 (PMC9769982; doi:10.1128/spectrum.02656-22)
Supplement: Supplemental file 1 — Fig. S1 and S2 and Table S1. Download spectrum.02656-22-s0001.pdf, PDF file, 2.0 MB [file spectrum.02656-22-s0001.pdf]

Supplemental Materials:
Increased rate of yeast cultivation from packaged beer with environmentally relevant anaerobic handling.

Kira Pai[1], Ginger Stout[1], Theresa Zimmer[1], Clayton Jacobs[1], Helene Ver Eecke,[1]#
[1]Metropolitan State University of Denver, Biology, Denver, CO, USA

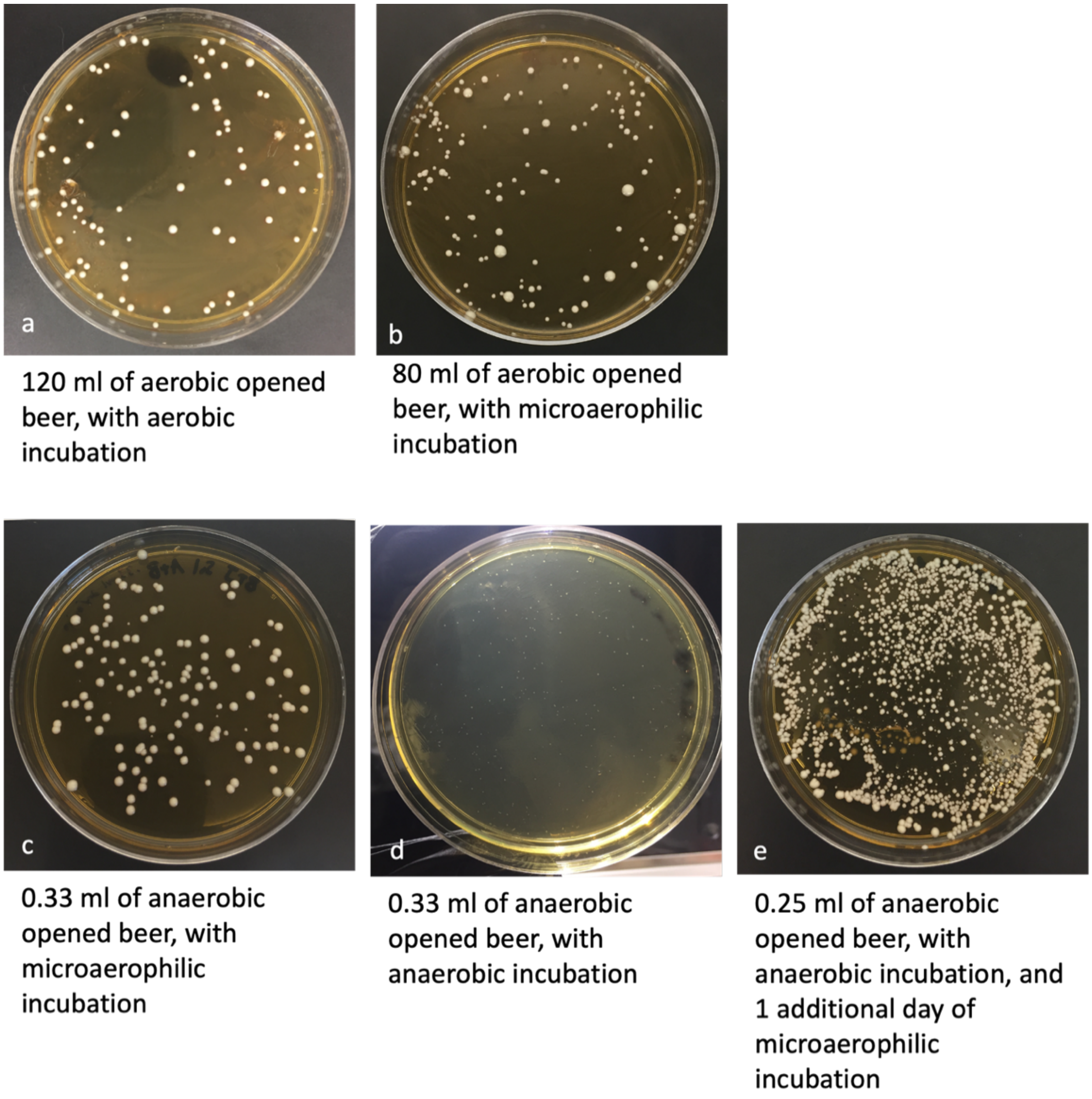

Figure S1

Representative images of 100 mm plates with yeast colonies recovered from concentrated Bamberger's Mahr's Bräu Unfiltered Lager with various oxygen handling methods during beer opening and plate incubation. Colonies were recovered after 4 days of aerobic incubation after

inoculation with 120 ml of concentrated beer opened aerobically (**a**). Colonies were recovered after 4 days of microaerophilic incubation after inoculation with 80 ml of concentrated beer opened aerobically (**b**). Colonies were recovered after 4 days of microaerophilic incubation after inoculation with 0.33 ml of concentrated beer opened anaerobically (**c**). Colonies were recovered after 4 days of anaerobic incubation after inoculation with 0.33 ml of concentrated beer opened anaerobically (**d**). Colonies were recovered after 1 day of microaerophilic incubation after 4 days of anaerobic incubation after inoculation with 0.25 ml of concentrated beer opened anaerobically (**e**). Colony size and count averages are summarized in **Figure 1** of primary manuscript.

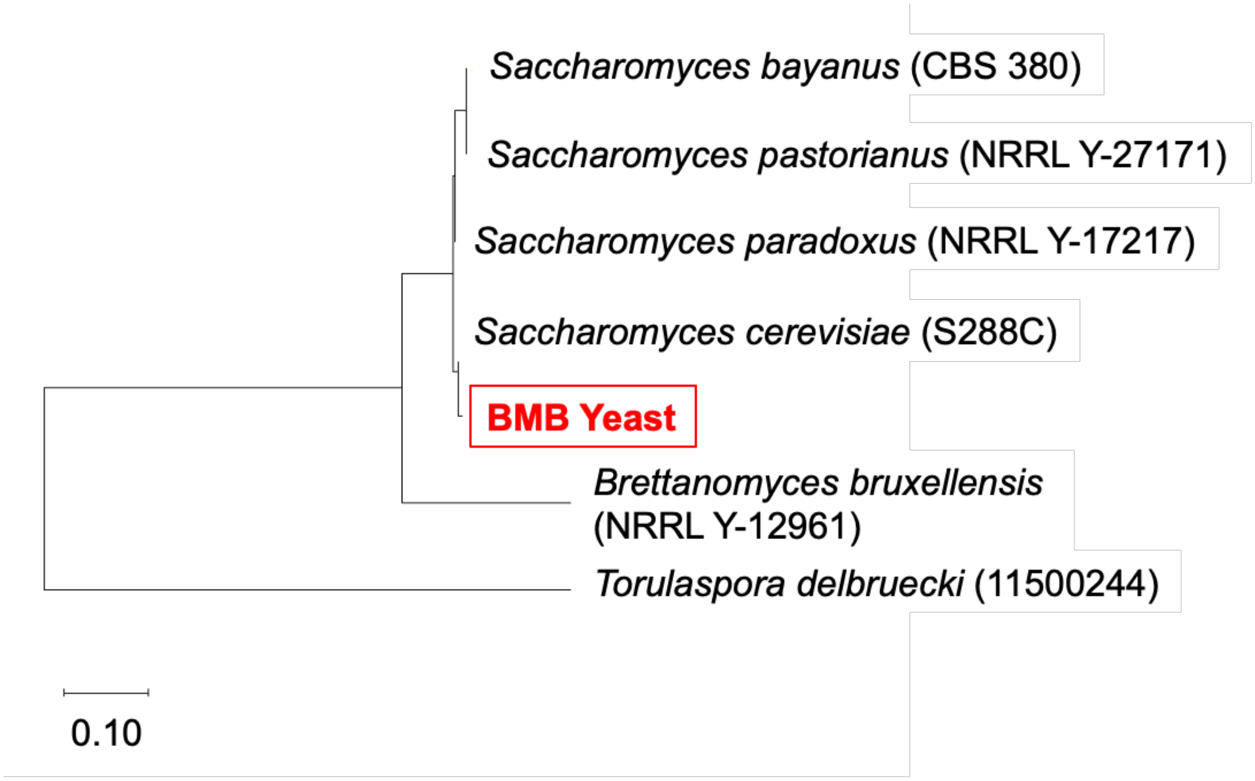

Figure S2

Neighbor joining phylogenetic relatedness of yeast enriched from Bamberger's Mahr's Bräu (BMB yeast) and common brewing yeasts based on taxonomic ribosomal gene sequencing.

Table S1

Select parameters of Bamberger’s Mahr’s Bräu Unfiltered Lager (BMB) packaged beer as measured with an Anton Paar Packaged Beer Analyzer (PBA – Beer Generation M - DMA4500MEC) with a carbo QC ME piercing unit All data is reported as AV (±SD) n=3.

Alcohol (% v/v)	Alcohol ASBC (%w/w)	Specific Gravity (CO_2-corr.)	Calories (kcal/12oz)	CO_2 Concentration (vol.)	O_2 Concentration (ppb)	Present Gravity
5.38 (±0)	4.22 (±0)	1.01 (±6e^{-6})	163.15 (±0.02)	2.99 (± 0.004)	0 (±0)	8.89 (±0.006)
